# Supplementary material for: The Intricate Role of Ecdysis Triggering Hormone Signaling in Insect Development and Reproductive Regulation
Source: Insects. 2023 Aug 16;14(8):711. doi: 10.3390/insects14080711 (PMC10455322; doi:10.3390/insects14080711)
Supplement: Supplementary file 1 [file insects-14-00711-s001.zip › insects-2499207-supplementary.pdf]

Table S1: Comparison of ETH signaling cascades and regulations between holo- and hemimetabolous insects.

Insect groups	Growth Stages	Componets and cascades of ETH signaling in Holometabolous insects	Componets and cascades of ETH signaling in Hemimetabolous insects
Holometabola	Egg-larval instars-pupa-adult (four distinct and typical phases of life cycle)	1) In most holometabolous insects (e.g., Lepidoptera, Diptera, and some Coleoptera and Hymenoptera) paired and large Inka cells are attached to tracheae in each prothoracic and abdominal segment.	1) In other holometabolous insects (e.g., beetles and bees) and all hemimetabolous insects, ETH is released from numerous small and scattered Inka cells dispersed throughout the tracheal system
		2) In all the holometabolous insects tested, Inka cells were found to present in 1-2-week-old adults.	2) In hemimetabolous insects Inka cells seem to degenerate after the emergence of adults.
		3) The mechanism of ETH signaling regulation and function on ecdysis has been well investigated in holometabolous insects (with complete metamorphosis, which includes four life stages: egg, larva, pupa, and imago or adult).	3) In contrast, very little information is available about the role of ETH signaling regulation and function in sequential and gradual growth and developmental changes associated with ecdysis in hemimetabolous insects.
Hemimetabola	Egg-nymphal instars-adult (developmental characteristics distingusihed with gradual changes)	4) The mechanism of ETH signaling regulation has been extensively studies in holometabolous insects including their specific underlined differences among species and at various stages of life cycles.	4) In hemimetabolous insects, the mechanism of ETH regulation has not been studied very well among species and during different stages of life cycle.
		5) ETH signaling plays a crucial role for the larva-larva ecdysis as well as in larva-pupa transition in holometabolous insects.	5) ETH signaling is associated with sequential and gradual growth and developmental changes in hemimetabolous insects.

		6) In holometabolous insects, ETH plays crucial role in regulating complex signaling cascade associated with insect development and reproductive regulation.<br><br>7) Most of the important genes/factors associated with ETH signaling cascades regulating insect development and reproductive success have been identified and well-studied in holometabolous insects.<br><br>8) In holometabolous insects, ecdysteroids from prothoracic gland play crucial role in the regulation of ovarian maturation and Oogenesis (e.g., at the onset of vitellogenesis) during adult stage, development of ovaries during pupal and pharate stages and niche and primordial germ cell differentiation during late larval stages.<br><br>9) 20E, ETH and JH hormonal triad network are crucial for reproductive success in several holometabolous adult insects including (e.g., *D. melanogaster* male and female). Various environmental stress responses, silencing of ETHR on CA through RNAi or conditional Inka cell ablation hampers reproductive success in adult insects *D. melanogaster*.<br><br>e.g., Fruit fly (*Drosophila melanogaster*), moths *(Bombyx mori* and *Manduca sexta)*, and yellow fever mosquito (*Aedes aegypti*), beetle *Tribolium castaneum etc.* [11, 44, 26].	6) ETH very little information is available in the role of ETH in insect development (indicated by gradual changes) and reproductive success.<br><br>7) Most of the crucial factors/gene-functions associated with intricate ETH signaling cascades regulating growth and reproductive regulations are yet to be identified in holometabolous insects.<br><br>8) While, in the hemimetabolous insects JH acts as the primary regulator of ovarian development and vitellogenin synthesis in the fat body. The titers of the ecdysteroids increase largely at the end of oogenesis and signals the termination of oogenesis (by terminating vitellogenesis).<br><br>9) Very little is known about the role of ETH in regulating JH biosynthesis in hemimetabolous adult insects. Only recently, low levels of SchgrETHR and the precursor of SchgrETH have been reported from the corpora allata-corpora cardiaca (CA-CC) complex of the desert locust, *Schistocerca gregaria*.<br><br>e.g., Desert locust (*Schistocerca gregaria*) Cockroach species (*Blattella germanica* and *Diploptera punctata*) etc. [25, 75].
